# Oropharyngeal meningococcal carriage in children and adolescents, a single center study in Buenos Aires, Argentina

**DOI:** 10.1371/journal.pone.0247991

**Published:** 2021-03-29

**Authors:** Angela Gentile, Maria Paula Della Latta, Mercedes Bloch, Luisina Martorelli, Barbara Wisner, Cecilia Sorhouet Pereira, Mabel Regueira, Maria del Valle Juarez, Veronica Umido, Adriana Efron

**Affiliations:** 1 Epidemiology Division, Dr Ricardo Gutiérrez Children’s Hospital, Buenos Aires, Argentina; 2 Clinical Microbiology Service, INEI-ANLIS Dr. Carlos G. Malbrán, Buenos Aires, Argentina; Public Health England, UNITED KINGDOM

## Abstract

**Background:**

*Neisseria meningitidis* (Nm) pharyngeal carriage is a necessary condition for invasive disease. We present the first carriage study in children in Buenos Aires, Argentina, considering 2017 as a transition year. Aims: to assess the rate of Nm carriage, to determine genogroup, clonal complex and outer membrane protein distribution, to determine carriage risk factors by age.

**Methods:**

Cross-sectional study including children 1–17 yrs, at Ricardo Gutiérrez Children’s Hospital in Buenos Aires 2017. Oro-pharyngeal swabs were taken and cultured within a short time after collection. Genogroup was determined by PCR and clonal complex by MLST. Categorical variables were analyzed.

**Results:**

A total of 1,751 children were included. Group 1: 943 children 1–9 yrs, 38 Nm were isolated; overall carriage 4.0%. Genogroup distribution: B 26.3%, W 5.3%, Y 2.6%, Z 5.3%, other groups 7.9% and capsule null (cnl) 52.6%. Participating in extracurricular activities was the only independent predictor of Nm carriage. Group 2: 808 children 10–17 yrs, 76 Nm were isolated; overall carriage 9.4%. Genogroup distribution: B 19.7%, C 5.3%, W 7.9%, Y 9.2%, Z 5.3%, other groups 7.9% and cnl 44.7%. Independent predictors of carriage: attending pubs/night clubs and passive smoking (adjusted OR: 0.55, 95%CI = 0.32–0.93; p = 0.025).

**Conclusions:**

Overall carriage was higher in 10–17 yrs. The isolates presenting the *cnl* locus were prevalent in both age groups and genogroup B was the second most frequent.

## Introduction

*Neisseria meningitidis* (*Nm*) is a leading cause of invasive diseases such as meningitis and septicaemia. Twelve different serogroups have been defined based on chemical composition and immunological specificity of capsular polysaccharides (A, B, C, E, H, I, K, L, W, X, Y, Z); most cases of invasive disease are caused by serogroups A, B, C, W, X and Y. Meningococci can be subdivided into serotypes and serosubtypes based on outer membrane proteins PorB and PorA respectively. In addition, immunotypes have been described dependant on the antigenic composition of the lipopolysaccharide [1]. Different groups of related sequence types, known as clonal complexes (CC) and hypervirulent lineages have been identified using molecular biology techniques [2–4].

While humans are the only known reservoir of *Nm*, meningococci are generally commensal organisms colonizing the nasopharynx, a phenomenon known as carriage, which can be transient or evolve into invasive meningococcal disease (IMD). Carriage is necessary for IMD to occur [5]. Some studies report asymptomatic carriage triggers an immune response providing protection [6]. Better understanding of factors influencing carriage is crucial to clarify disease dynamics like occur with conjugate polysaccharide vaccines, that can impact carriage and contribute to population immunity. Carriage prevalence increases with age, rates ranging from 4.5% in children to 23.7% in adolescents have been reported worldwide; children over 10 years of age, adolescents and young adults are the main reservoirs of *Nm* and are mostly responsible for transmission [7,8].

Meningococcal carriage rates are higher in closed or semi/enclosed communities (military personnel, university students), and in close contacts of patients with IMD. Several risk factors have been associated with carriage including cigarette smoking, exposure to passive smoking, intimate kissing and certain social behaviours such as frequenting pubs or nightclubs [9–11].

Global incidence of IMD in Argentina ranges from 0.4 to 0.7 cases per 100,000 population per year, mainly affecting children under the age of 5 years. Highest rates are observed in infants under 12 months (13.2 cases per 100,000), 64% of whom are under 9 months. The incidence of IMD in Argentine, does not increase in adolescents [12].

Genogroups B and W are the main cause of invasive disease in the country, and corresponded to 91% of all clinical isolates between 2012 and 2015. Genogroup B proportion has increased substantially in recent years accounting for 57% of all samples analysed at the National Reference Laboratory (NRL) for Meningitis and Acute Bacterial Respiratory Infections INEI-ANLIS C. G. Malbrán in 2017. Other common genogroups were W (25.3%), C (11.4%) and Y (6.3%) [13–15].

In January 2017, the quadrivalent meningococcal conjugate vaccine MenACYW (conjugated to the non-toxic CRM_197_ derivative of diphtheria toxin) was included in the National Immunization Program (NIP), as a combined strategy targeting both infants (2+1 doses at 3, 5 and 15 months) and adolescents (single dose at 11 years). We consider 2017 as a transition year, since in that moment the strategy began with infants aged 3 and 5 months and in adolescents coverage was less than 50%. This study was carried out before local regulatory authorities had approved meningococcal B vaccine use [16].

The aim of this study was to assess *Nm* oropharyngeal carriage rates in children and adolescents attending a public, tertiary children’s hospital in the city of Buenos Aires, analyse risk factors in different age groups and determine circulating serogroups, clonal complexes and outer membrane protein distribution.

## Materials and methods

Between March and December 2017, we carried out an observational, cross-sectional study in 1751 children and adolescents aged 1 to 17 years old at the Ricardo Gutiérrez Children’s Hospital in Buenos Aires city. Participants were distributed into two age groups: 1 to 9 years (group 1, n = 943) and 10 to 17 years old (group 2, n = 808). The sample size was chosen based on an estimated meningococcal carriage rate of 2.5% (95% confidence interval (CI) 1.5–3.5) for the 1 to 9 year-olds and 5% (95% CI 3.5–6.5) for the older children. At the moment of the design of our study, we hoped to obtain at least 100 Nm samples positive to be analysed. Based on previous Nm carriage studies carried on in Argentina, we calculated to need 1740 samples to get this 100 Nm to study.

### Inclusion criteria

Children and adolescents aged 1 to 17 years attending the outpatient clinic at the Ricardo Gutiérrez Children’s Hospital in Buenos Aires.

### Exclusion criteria

Subjects with fever at the time of swab collection, immunodeficiency disorders (congenital, acquired, or secondary immune disorders due to treatment, cancer, Down Syndrome), prior meningococcal conjugate vaccination and patients receiving antimicrobial treatment at the time of the study or in the past 24 hours were excluded.

### Subject recruitment

A convenient sampling was carried out. Every subject attending to the hospital was invited to participate in the study, and asked to sign an informed consent/assent form before swabs were collected. In all cases, children were enrolled in the presence of their parents or guardians, who also signed an informed consent/assent form.

### Data collection

A questionnaire was used to collect data on: demographics (age, gender, place of residence); crowded housing conditions; maternal educational level (partial or complete primary studies; partial or complete secondary studies; partial or complete university studies); cigarette smoking; exposure to passive smoking; school attendance (kindergarten, school); extracurricular activities (frequent -2 or more days in a week- contact with other children in sports/arts or other activities outside the school); pub/nightclub attendance. (S1 File).

Participants were considered carriers when nasopharyngeal swab cultures were positive for *Nm* (excluding other *Neisseria sp*). Data were collected anonymously, privacy rights of patients were observed in all cases in accordance with local legislation and the World Medical Association Declaration of Helsinki International Code of Ethics for experiments involving humans. The protocol and informed consent form were approved by the Ricardo Gutierrez Children Hospital Independent Committee on Ethics in Investigation.

### Isolation and characterization of N. meningitidis

Oro-pharyngeal swab samples were obtained through the mouth from the posterior wall of the oropharynx by trained staff, and immediately placed in Amies-Charcoal transport medium (Britania, Argentina). Samples transferred to the NRL within 5 hours of collection were plated onto modified Thayer-Martin medium (Britania, Argentina), incubated at 37°C in a humid atmosphere containing 5% CO2 and examined after 24 and 48 hours. Colonies characteristic of *Neisseria sp*. were sub-cultured on blood agar medium for species identification by Gram staining, oxidase reaction (Britania, Argentina), gamma-glutamyl aminopeptidase reaction (ROSCO, MEDICA-TEC) and Bruker Biotyper matrix-assisted laser desorption ionization-time of flight mass spectrometry (MALDI-TOF MS).

Isolates identified as *N*. *meningitidis* were confirmed by PCR using specific primers for detecting *crg*A gene, capsular transport gene *ctr*A and genogroups A, B, C, E, W, X, Y and Z [17–19]. Isolates negative for *crg*A and *ctr*A PCR were re-tested by PCR for presence of the capsule null region (*cnl*) [20].

To determine sequence type (ST) and clonal complex (CC), Multilocus Sequence Typing (MLST) was performed as previously described by Maiden et al., using primers listed on the Neisseria PubMLST website (http://pubmlst.org/neisseria) [3].

For the characterization of PorA genotype, Factor H-binding protein (fHbp) and neisserial heparin-binding antigen (NHBA), PCR amplification of variable region gene fragments followed by standard DNA sequencing was performed [21–24]. The assignment of genotype, variant family and peptide variant respectively was achieved using the blast tool of the Neisseria.org website (http://pubmlst.org/neisseria) [3]. The nomenclature scheme for fHbp peptide according to Novartis was used. For NHBA the peptides variants designations from Bexsero Antigen Sequence Typing (BAST) was the chosen nomenclature for show the results.

The presence of neisserial adhesion A (NadA) gene was determined by PCR amplification as previously describe [25].

All the primers used in this study for PCR and sequence analyses are listed in S1 Table.

### Statistical analysis

Categorical variables were analysed using the χ2 test with Yates correction and the Wilcoxon test was applied for median age comparison. Odds ratio (OR) with 95% CI was used for association analysis; a bivariate analysis was performed initially to identify significant associations and multivariable logistic regression subsequently carried out to establish independent predictors of *Nm* carriage. P values < 0.05 were considered statistically significant. STATA/SE version 13 was used for the analysis. A logistic regression model was constructed to identify predictors of *Nm* carriage. Variables significantly associated with carriage in the crude analysis and/or those considered clinically relevant, were added one at a time to the multivariable model and only those showing significant association with the outcome in the multivariable context (Wald test) were retained in the final model. Changes in coefficients were examined to find confounding variables. Model calibration and discrimination were evaluated using Hosmer-Lemeshow goodness-of-fit test and area under the ROC curve.

## Results

A total of 1751 children were enrolled, 943 1–9 years (Group 1) and 808 10–17 years (Group 2). Demographics and epidemiologic characteristics of each group are shown in Table 1.

**Table 1:** Characteristics of patients according to age groups (Group 1: 1–9 years and Group 2: 10–17 years).

Demographics and risk factors		Group 1 (n = 943)	Group 2 (n = 808)
Age	Median (years); IQR	5.3; 3.4–7.7	12.5; 11.1–14.2
Gender (male) (n, %)		481 (51)	399 (49.4)
Residence: Buenos Aires suburbs (n, %) Buenos Aires city Others		583 (61.9)	448 (55.4)
		256 (27.2)	233 (28.8)
		103 (10.9)	127 (15.7)
Crowded housing conditions (n, %)	Simple (up to 3 people per room)	283 (30)	206 (25.6)
	Critical (more than 3 people per room)	182 (19.3)	122 (15.1)
Maternal Educational Level*(n, %)	Partial primary	69 (7.32)	46 (5.7)
	Complete primary	171 (18.1)	207 (25.6)
	Partial secondary	232 (24.6)	185 (22.9)
	Complete secondary/partial tertiary	395 (41.9)	290 (35.9)
	Complete university	47 (5)	59 (7.3)
Passive smoking (n, %)		313 (33.2)	329 (40.7)
School attendance (Kindergarten, school) (n, %)		731 (77.5)	778 (96.3)
Participation in extracurricular activities (n, %)		237 (25.1)	370 (45.9)
Pub/nightclub visits (n,%)		-------	27 (3.3)

*Schooling: Primary (7 years schooling), secondary (11 years), tertiary/university (>11 years).

Nm carriage rate was 4% (n = 38) in group 1 and 9.4% (n = 76) in group 2 (S1 Fig). Nm carriage percentage increased with age (S2 Fig).

Multivariable analysis showed participating in extracurricular activities was associated with *Nm* carriage in group 1 (OR: 2.25; 95% CI: 1.16–4.36; p = 0.016). In group 2, visiting pubs/nightclubs was carriage associated (OR: 3.37; 95% CI: 1.39–9.98; p = 0.009), whereas passive smoking was not carriage associated (OR: 0.51; 95% CI: 0.30–0.89; p = 0.018) (Table 2). We performed a stratified analysis by age, dividing Group 2 into two subgroups. The cut-off point was established at 13 years, which is the age from which adolescents can go to pubs. In the subgroup aged 13 to 17 years (n = 343), the OR for the variable of attendance at pubs was 3 (CI 95% 1.1–8.4 p = 0.036).

**Table 2:** Results of multiple logistic regression: Independent predictors of *Nm* carriage according to age group.

Age Group	Independent predictors	Odds Ratio	95% CI	*P*
Group 1 (1-9ys)	Extracurricular activities	2.25	1.16–4.36	0.016
	Crowded housing conditions	1.43	0.74–2.76	0.28
	Maternal education (less than 11 years schooling)	0.62	0.32–1.22	0.17
	Passive smoking	1.03	0.52–2.05	0.11
	Prior use of antibiotics	0.55	0.19–1.57	0.26
	School attendance	2.53	0.88–7.23	0.08
Group 2 (10-17ys)	Extracurricular activities	1.33	0.81–2.18	0.25
	Crowded housing conditions	1.21	0.73–2.00	0.45
	Maternal education (less than 11 years schooling)	1.01	0.61–1.67	0.96
	Passive smoking	0.51	0.30–0.89	0.018
	Prior use of antibiotics	0.48	0.18–1.28	0.14
	School attendance	0.59	0.19–1.80	0.36
	Pub/nightclub visits	3.73	1.39–9.98	0.009

### Genogroups

The isolates harboring the *cnl* locus were predominant in both age groups followed by genogroup B. A total of 38 isolates were identified in Group 1: cnl 52.6% (20), B 26.3% (10), other groups 7.9% (3), W 5.3% (2), Z 5.3% (2), and Y 2.6% (1). In group 2, 76 isolates were found: cnl 44.7% (34), B 19.7% (15), Y 9.2% (7), W 7.9% (6), other groups 7.9% (6), C 5.3% (4), and Z 5.3% (4).

### Clonal complexes

Clonal complexes were determined in 32 of 38 *Nm* isolates in group 1 and in 73 of 76 isolates in group 2. In both age groups, ST-198 and ST-1136 CCs were identified only among *cnl* isolates, ST-865 and ST-32 were identified only among genogroup B and ST-11 only among genogroup W. ST 41–44 was most often associated with genogroup B (S3 and S4 Figs).

### Outer membrane protein sequencing

PorA was identified in 87 out of 105 isolates; one variable region sequence was determined in 13 isolates. Due to the high degree of heterogeneity among genogroups, differentiation by age group was not possible. The isolates presenting *cnl* locus were mainly associated with porA 22,14 (13/47) (S5 Fig). As regards genogroup B, the number of isolates was too low to identify any porA predominance (S6 Fig). In the ST-198 CC, porA 22,14 predominated (12/31), in ST-35 CC porA 22–1,14 predominated (8/11), in ST-865 only porA 21,16–36 was identified (3/3), and ST-11 was only associated with porA 5,2 (3/3).

We were able to identify fHbp peptides variant in 22 isolates belonging to group 1 (68.8%) and in 65 belonging to group 2 (89.0%); fHbp variant family 2 predominated in both. ST-198 CC was only associated with fHbp variant family 1; ST-41/44 and ST-865 CCs were associated with fHbp variant family 2. A single group 2 isolate expressed fHbp variant family 3 (ST-461, genogroup C) (S7 and S8 Figs).

NHBA was determined in 67 isolates, 14 in group 1(43.8%) and 51 in group 2 (69.9%). A high degree of variability in NHBA variants was found, 23 different peptides were identified. ST-198 was associated with peptide 10 (4/7 group 1 and 18/24 group 2), ST-35 with peptide 21 (1/3 group 1 and 6/8 group 2), ST-1136 with peptide 145 and ST-175 with peptide 9.

NadA was identified in 10.5% (n:4) of isolates in group 1 (B:2, W:1, Z:1) and in 9.6% (n:7) of isolates in group 2 (Y:3, B:2, W:1, NG:1).

The isolate information of molecular data is provided in S2 Table.

## Discussion

This was the first study of *Nm* oropharyngeal carriage in children and adolescents conducted in Argentina before introduction of the quadrivalent meningococcal conjugate vaccine to the NIP. Collecting baseline data is crucial to make decisions based on the local epidemiology, since carriage of serogroups and CCs varies by geographic regions [26]. Impact of immunization on invasive disease and carriage rates after introduction of new conjugate vaccines is also important to measure. In the United Kingdom, two years after MenC conjugate vaccine was used in the national immunization program, carriage prevalence in adolescents were reduced from 0.98% to 0.5%, confirming vaccine vaccine effect on acquisition of carriage strains [27].

Carriage prevalence varies with age, epidemiology of circulating *Nm* and local vaccination policies. In Europe and in USA, rates of carriage are very low in infants and young children increasing substantially in adolescents [28]. Several studies conducted in Latin America have shown varying results between countries. In Cuba, Martinez et al found highest *Nm* carriage rates in children over the age of 9 years, peaking in 11 year-olds (36.4%) [7]. In Brazil, carriage prevalence in 2015 was 9.9% in adolescents between 11 and 19 years of age [29]. Rates reported in Mexico were lower, 1.9% in children and 2.9% in adolescents [30].

In this study, the population was divided into two age groups, below or above the age of 10 years, based on evidence that carriage was higher in adolescents and taking into account that the classification of WHO, define the beginning of the adolescence at this age. We found a higher prevalence rate in adolescents (9.4%), similar to those reported in a recent meta-analysis of *Nm* carriage in the American continent (approximately 9% prevalence in this age group in the Southern Cone region) [26].

Several risk factors have been associated with meningococcal carriage including male gender, number of individuals living together, parental educational level, exposure to cigarette smoke, attending pubs/nightclubs [11,26,31,32]. In our study, pub/nightclub visits in adolescents and participation in extracurricular recreational activities in 1–9 year-olds were significantly associated with increased risk of being a carrier, a finding consistent with existing knowledge that close personal contact is linked to carriage risk. No association was found between other risk factors (high number of household members, parental educational level, school attendance, history of recent antibiotic use) and carriage.

Although passive smoking is often associated with carriage, we found mixed evidence for the effect of passive smoking on carriage [10,33]. Smoke exposure is a known risk factor for developing IMD, however the relationship with carriage is less clear since some studies have not found an association between passive smoking and carriage [34]. Several microbiological hypotheses help explain the mechanisms by which cigarette smoke damages the nasopharyngeal mucosa, thus affecting carriage. Smoke inhibits neutrophil phagocytic activity, dampens natural killer cell cytotoxicity, reduces immunoglobulin A secretion, induces oxidative stress and has a negative effect on immune response including response to vaccination. Passive coating of the oropharyngeal mucosa with tobacco smoke components can potentially enhance binding of pathogenic bacteria, including meningococci [33,35,36].

Some authors believe that increased risk of carriage in children exposed to passive smoking is due to close contact with the smoker rather than to effects of the smoke per se [29]. However, studies are often biased as information is provided by parents and may be incorrect; further investigations measuring nicotine levels are needed to clarify this issue.

In concordance with other similar regional reports, *cnl* meningococci was the most common isolate identified [26,38]. Genogroup B was the second most prevalent as our previous study [38]. Predominance of *cnl* strains has been widely reported in healthy carriers [37]. Clonal complexes associated with *cnl* isolates were ST-198, as previously described in healthy carriers in Argentina, and to a lesser degree ST-1136, which were only found in 1–9 year-olds [38]. On analysing the antigenic composition of ST-198 CC, we observed a homogeneous distribution of surface proteins variants.

Genogroup B was the second most predominant genogroup in both age groups, coinciding with reports of prevalent genogroup causing meningococcal disease in the country [13–15]. Clonal complexes among genogroup B meningococci from carriage samples were the same CCs as those identified in IMD in Argentina in previous studies [39,40]. This genogroup harbored various CCs and outer membrane proteins. This variability reflects local epidemiological conditions characterised by a low number of cases per year and diverse antigenic profile of circulating disease isolates [39].

In a prior study carried out in Argentina, genogroup B ST-865 CC was prevalent in IMD; even if this CC was found to cause sporadic cases of meningococcal disease in other countries, in our country it behaves as predominant for many years (2006–2011) in genogroup B [39,40]. The situation remains the same for the period 2012–2014 (unpublished data). In this study, only three isolates belonging to this CC were identified, two in the younger age group and one in the older group.

All three isolates presented characteristic surface proteins, porA 21,16–36 and fHbp variant family 3, and did not exhibit NadA. Eight isolates belonging to ST-41/44, six belonging to ST-35, and 5 belonging to ST-32 were found, antigenic profiles were heterogeneous. ST-41/44 and ST-32 complexes exhibited a high diversity of porA antigens, whereas ST-35 presented mainly porA 22–1,14, as found in isolates from IMD. With regard to fHbp peptides, all three CCs harbored variant family 2 exclusively. NadA gene was found solely in ST-32 CC. Finally, although NHBA presence was variable, ST-35 CC presented mainly peptide 21. Antigenic diversity of genogroup B in healthy carriers coincided with endemic IMD epidemiology in Argentina.

Genogroup W carriage rate was low and the hypervirulent, hyperinvasive ST-11 CC was detected, only 1 isolate in group 1 and 2 isolates in group 2 (3.1% and 2.7% respectively). In Chile, an extensive carriage study was recently conducted after an outbreak of serogroup W meningococcal disease with high case-fatality rate. Although overall prevalence of genogroup W carriage was low, hyperinvasive ST-11 was the only clone identified [41,42]. In this study, genogroup W showed a variable antigenic profile; less than half of isolates belonged to ST-11, and harboured porA 5.2, fHbp variant family 2 and the NadA gene, similar to strains isolated from invasive disease cases in the country [39]. The remaining genogroup W isolates belonged to various CCs, mainly-ST-35 which was also found in genogroup B.

The B:ST-865 clone and the hyperinvasive W: ST-11clone were already found in a low proportion in previous studies [38]. The reason why these clones are found in low proportion in healthy carriers is still unclear. It may be due to their virulent characteristics, but further studies would be necessary to answer this question.

Despite not being able to analyse 100% of the sequences of the most relevant protein antigens, we believe that these results, although partial, enrich the knowledge of the description of the clones present in carriage in our environment. For future studies it would be very useful to have more tools for the performance of the characterization of the isolates.

Other genogroups were isolated in lower numbers, namely C, Y and Z, showing variable CCs and outer membrane proteins. Although genogroup Z is not a cause of IMD in Argentina, this strain was identified in healthy carriers. Circulating CCs associated in carriers with genogroup Y were different to those found in isolates causing IMD, in which ST-167 predominated [39,43].

Isolates of genogroup C were heterogeneous as seen in prior carriage and invasive disease studies; no group C carriage was observed in young children, but 5% in older children [38,39]. Only one isolate of clonal complex ST-103 was detected, although this clone is the main cause of invasive disease in neighbouring Brazil [29,44].

One of the main limitations to this study is the fact that the population included children attending an outpatient clinic at a public tertiary children’s hospital in Buenos Aires City, which may not be representative of country population. To minimize this bias, we only selected participants without the previously described immunosuppressing disorders. Other limitation was that the sample size did not allow stratified analysis within the defined groups. We must mention that the heterogeneity within the groups could be a limitation because the groups contain a large number of age groups with different social, immunological and developmental stages, and therefore there may be confounding factors. Other measures to minimize the bias of detection were taken: samples were plated promptly, PCR was used to identify genogroups, vaccinated children were excluded and herd immunity had not yet been achieved during the year of the vaccine introduction (2017).

In conclusion *N*. *meningitidis* carrier rates were higher among 10 to 17 year olds. The isolates containing the *cnl* locus were predominant in both age groups and genogroup B was the most common capsulated strain. Extracurricular activities in children and frequenting pubs/nightclubs in adolescents showed positive correlation with *Nm* carriage. Association between CC and outer membrane proteins in genogroups B and W coincide with those of IMD for the country. Genogroup Z was detected, although no cases of invasive disease have been reported in this country.

## Supporting information

S1 Fig Distribution of *Neisseria* species according to age group. (PPTX)

S2 Fig Samples (Nm positive, Nm negative) and Nm carriage percentage by age. (PPTX)

S3 Fig Distribution of clonal complexes by genogroup in the 1–9 year old group. (PPTX)

S4 Fig Distribution of clonal complexes by genogroup in the 10–17 year old group. (PPTX)

S5 Fig Distribution of PorA types in cnl isolates. (PPTX)

S6 Fig Distribution of PorA types in genogroup B isolates. (PPTX)

S7 Fig Distribution of fHbp variant families by clonal complex in the 1–9 year old group. (PPTX)

S8 Fig Distribution of fHbp variant families by clonal complex in the 10–17 year old group. (PPTX)

S1 Table Oligonucleotides used in *N*. *meningitidis* molecular typing. (DOCX)

S2 Table Molecular data. (DOCX)

S1 File (DOCX)
