# Supplementary material for: Oropharyngeal meningococcal carriage in children and adolescents, a single center study in Buenos Aires, Argentina
Source: PLoS One. 2021 Mar 29;16(3):e0247991. doi: 10.1371/journal.pone.0247991 (PMC8006983; doi:10.1371/journal.pone.0247991)
Supplement: S1 Fig — (PPTX) [file pone.0247991.s001.pptx]

## Slide 1
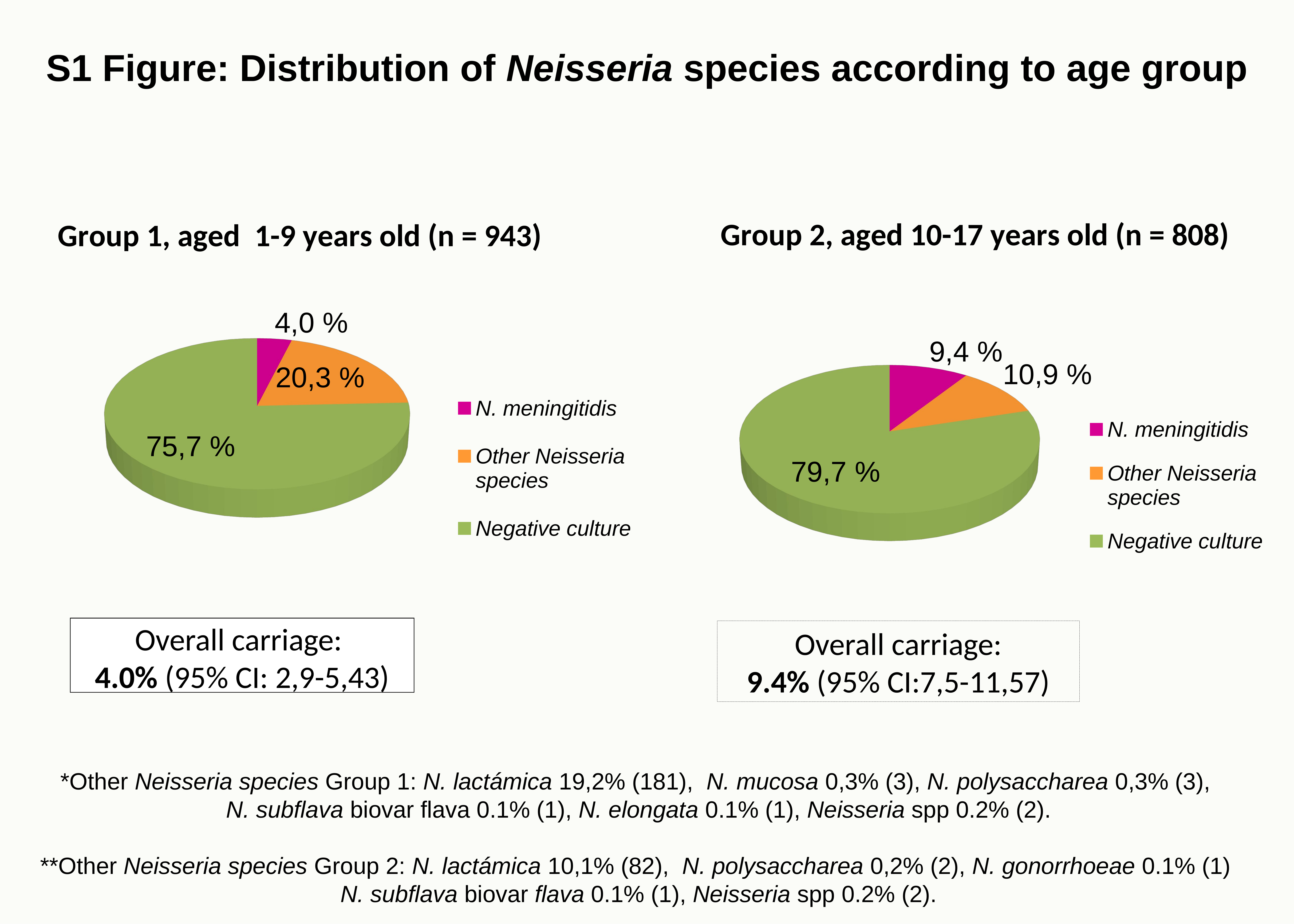

S1 Figure: Distribution of Neisseria species according to age group
[unsupported chart]
[unsupported chart]
Overall carriage:
4.0% (95% CI: 2,9-5,43)
Overall carriage:
 9.4% (95% CI:7,5-11,57)
*Other Neisseria species Group 1: N. lactámica 19,2% (181), N. mucosa 0,3% (3), N. polysaccharea 0,3% (3),
N. subflava biovar flava 0.1% (1), N. elongata 0.1% (1), Neisseria spp 0.2% (2).
**Other Neisseria species Group 2: N. lactámica 10,1% (82), N. polysaccharea 0,2% (2), N. gonorrhoeae 0.1% (1)
N. subflava biovar flava 0.1% (1), Neisseria spp 0.2% (2).
