# Supplementary material for: Oropharyngeal meningococcal carriage in children and adolescents, a single center study in Buenos Aires, Argentina
Source: PLoS One. 2021 Mar 29;16(3):e0247991. doi: 10.1371/journal.pone.0247991 (PMC8006983; doi:10.1371/journal.pone.0247991)
Supplement: S2 Fig — (PPTX) [file pone.0247991.s002.pptx]

## Slide 1
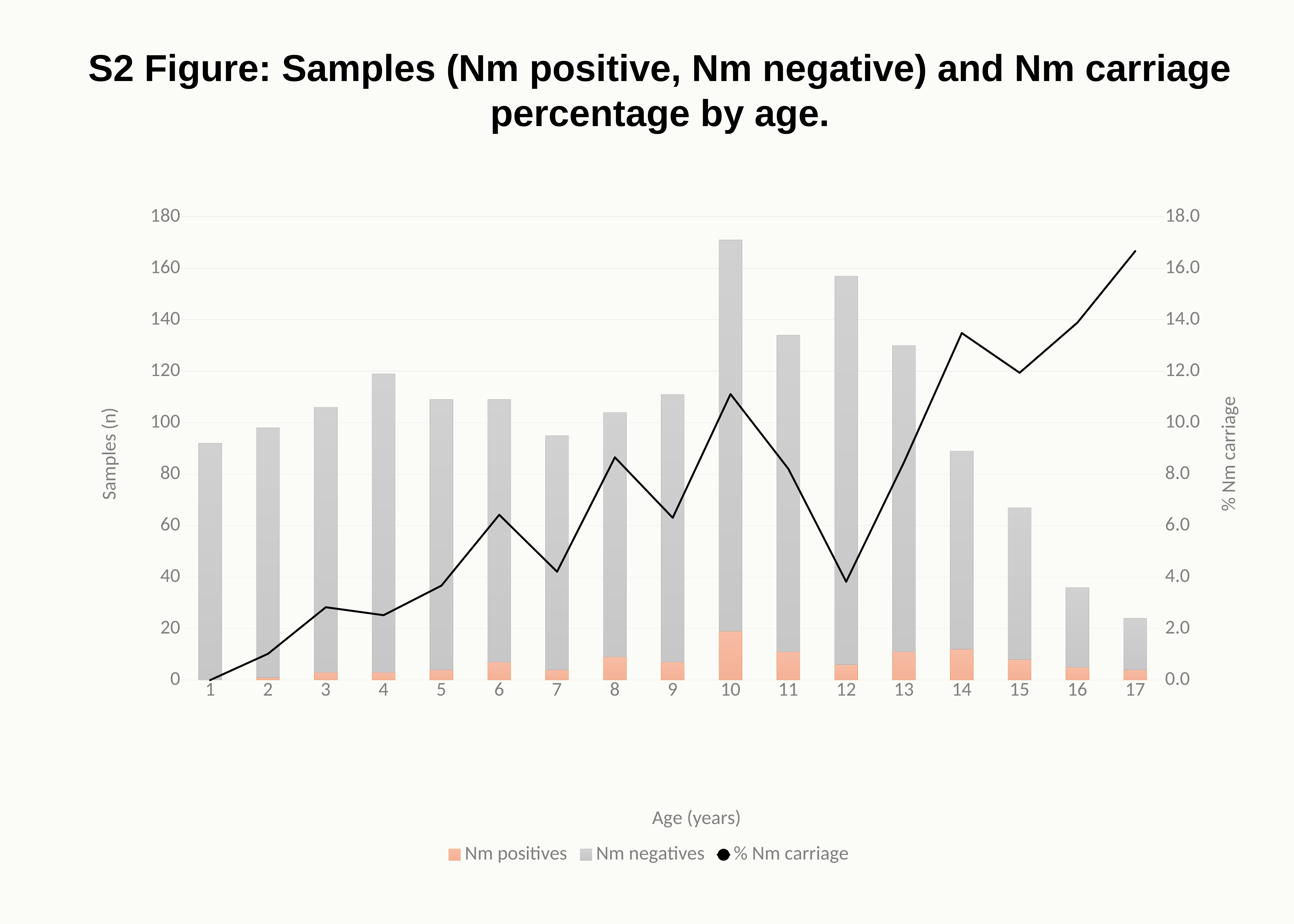

S2 Figure: Samples (Nm positive, Nm negative) and Nm carriage percentage by age.
### Chart
| Category | Nm positives | Nm negatives | % Nm carriage |
|---|---|---|---|
