# Supplementary material for: Oropharyngeal meningococcal carriage in children and adolescents, a single center study in Buenos Aires, Argentina
Source: PLoS One. 2021 Mar 29;16(3):e0247991. doi: 10.1371/journal.pone.0247991 (PMC8006983; doi:10.1371/journal.pone.0247991)
Supplement: S3 Fig — (PPTX) [file pone.0247991.s003.pptx]

## Slide 1
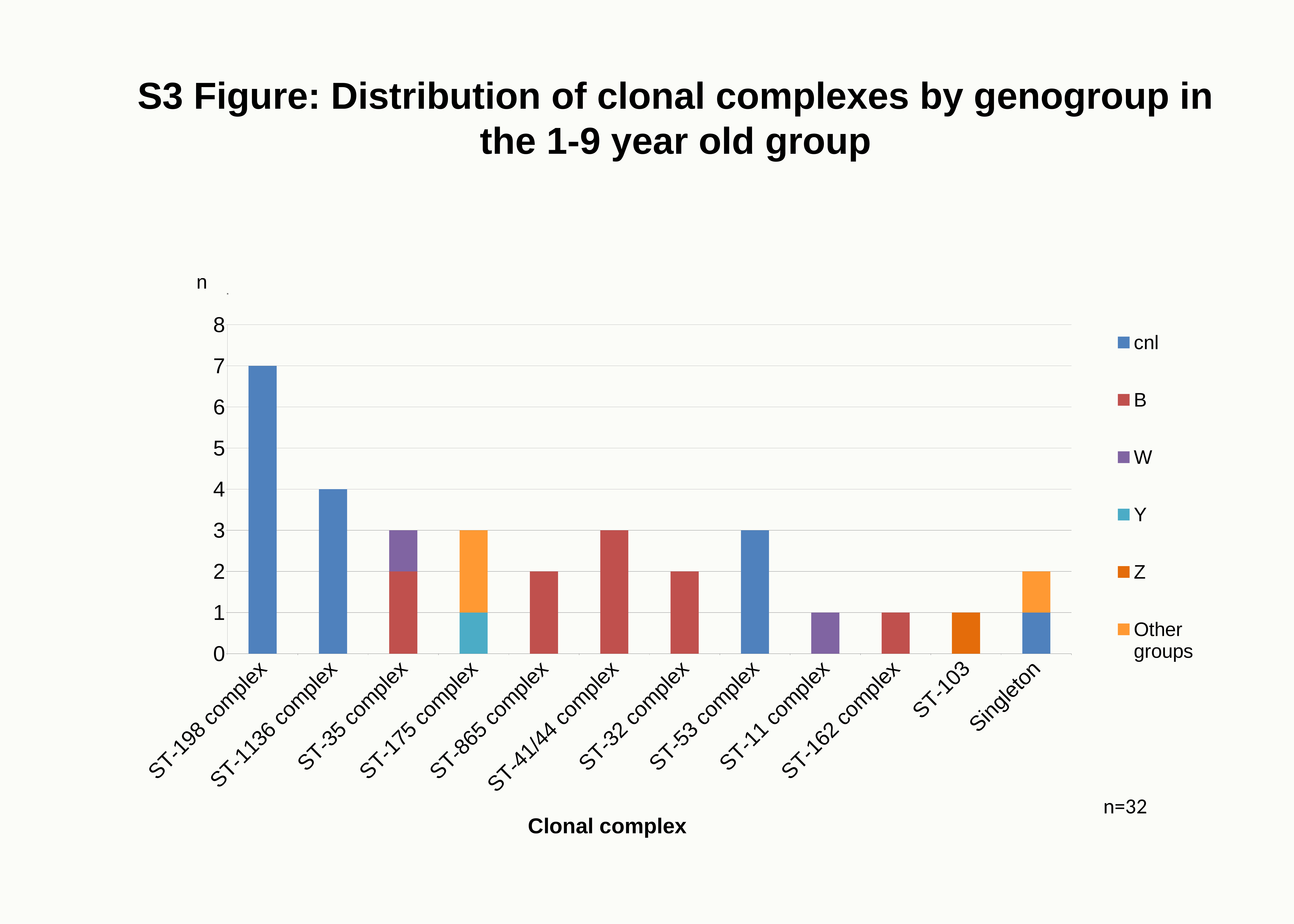

S3 Figure: Distribution of clonal complexes by genogroup in the 1-9 year old group
### Chart
| Category | cnl | B | W | Y | Z | Other groups |
|---|---|---|---|---|---|---|
| ST-198 complex | 7.0 | None | None | None | None | None |
| ST-1136 complex | 4.0 | None | None | None | None | None |
| ST-35 complex | None | 2.0 | 1.0 | None | None | None |
| ST-175 complex | None | None | None | 1.0 | None | 2.0 |
| ST-865 complex | None | 2.0 | None | None | None | None |
| ST-41/44 complex | None | 3.0 | None | None | None | None |
| ST-32 complex | None | 2.0 | None | None | None | None |
| ST-53 complex | 3.0 | None | None | None | None | None |
| ST-11 complex | None | None | 1.0 | None | None | None |
| ST-162 complex | None | 1.0 | None | None | None | None |
| ST-103 | None | None | None | None | 1.0 | None |
| Singleton | 1.0 | 0.0 | 0.0 | 0.0 | 0.0 | 1.0 |n
