# Supplementary figures and images for: Oropharyngeal meningococcal carriage in children and adolescents, a single center study in Buenos Aires, Argentina
Source: PLoS One. 2021 Mar 29;16(3):e0247991. doi: 10.1371/journal.pone.0247991 (PMC8006983; doi:10.1371/journal.pone.0247991)

## Slide 1
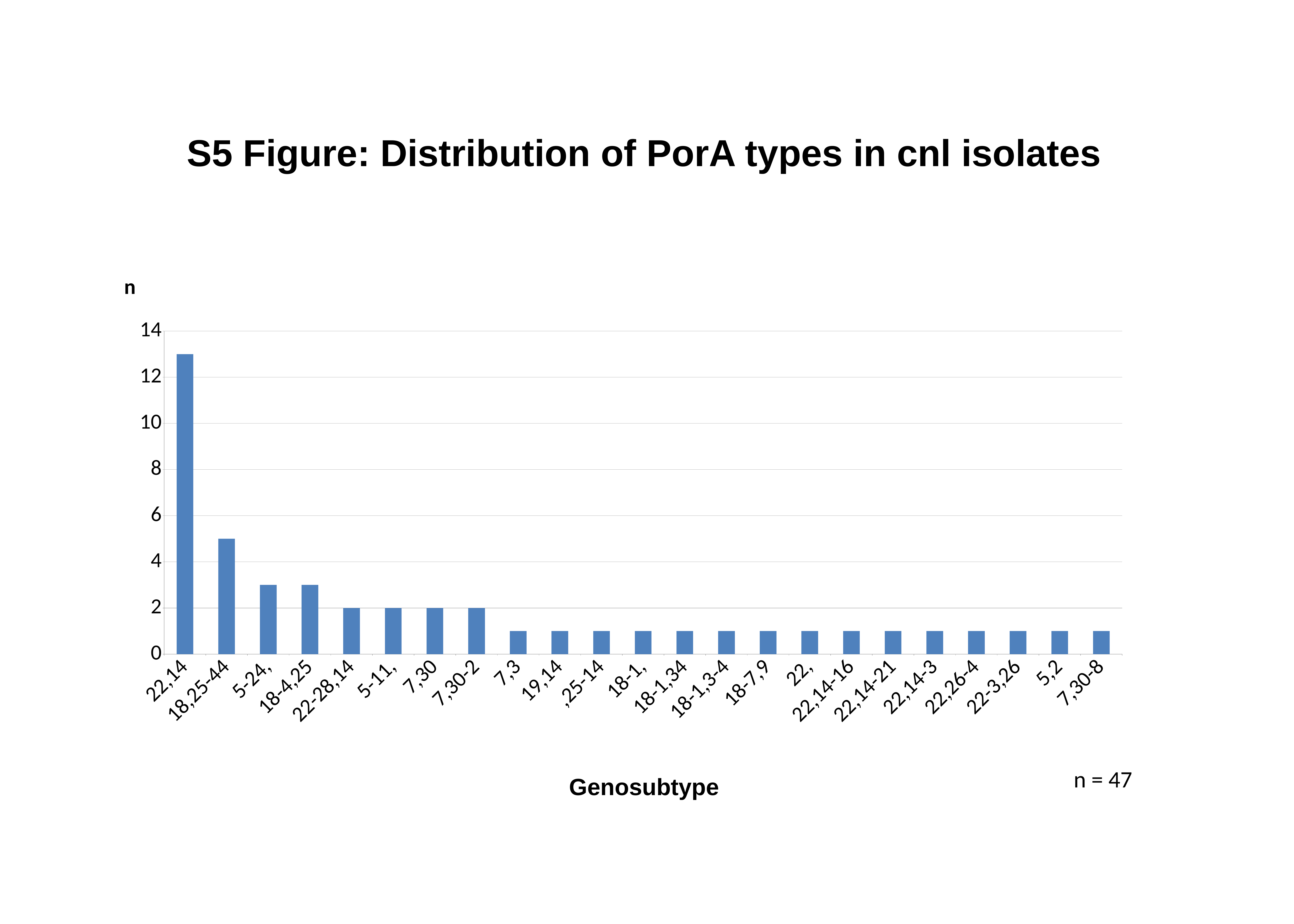

S5 Figure: Distribution of PorA types in cnl isolates
### Chart
| Category | |
|---|---|
| 22,14 | 13.0 |
| 18,25-44 | 5.0 |
| 5-24, | 3.0 |
| 18-4,25 | 3.0 |
| 22-28,14 | 2.0 |
| 5-11, | 2.0 |
| 7,30 | 2.0 |
| 7,30-2 | 2.0 |
| 7,3 | 1.0 |
| 19,14 | 1.0 |
| ,25-14 | 1.0 |
| 18-1, | 1.0 |
| 18-1,34 | 1.0 |
| 18-1,3-4 | 1.0 |
| 18-7,9 | 1.0 |
| 22, | 1.0 |
| 22,14-16 | 1.0 |
| 22,14-21 | 1.0 |
| 22,14-3 | 1.0 |
| 22,26-4 | 1.0 |
| 22-3,26 | 1.0 |
| 5,2 | 1.0 |
| 7,30-8 | 1.0 |Genosubtype

Supplement: S5 Fig — (PPTX) [file pone.0247991.s005.pptx]

## Slide 1
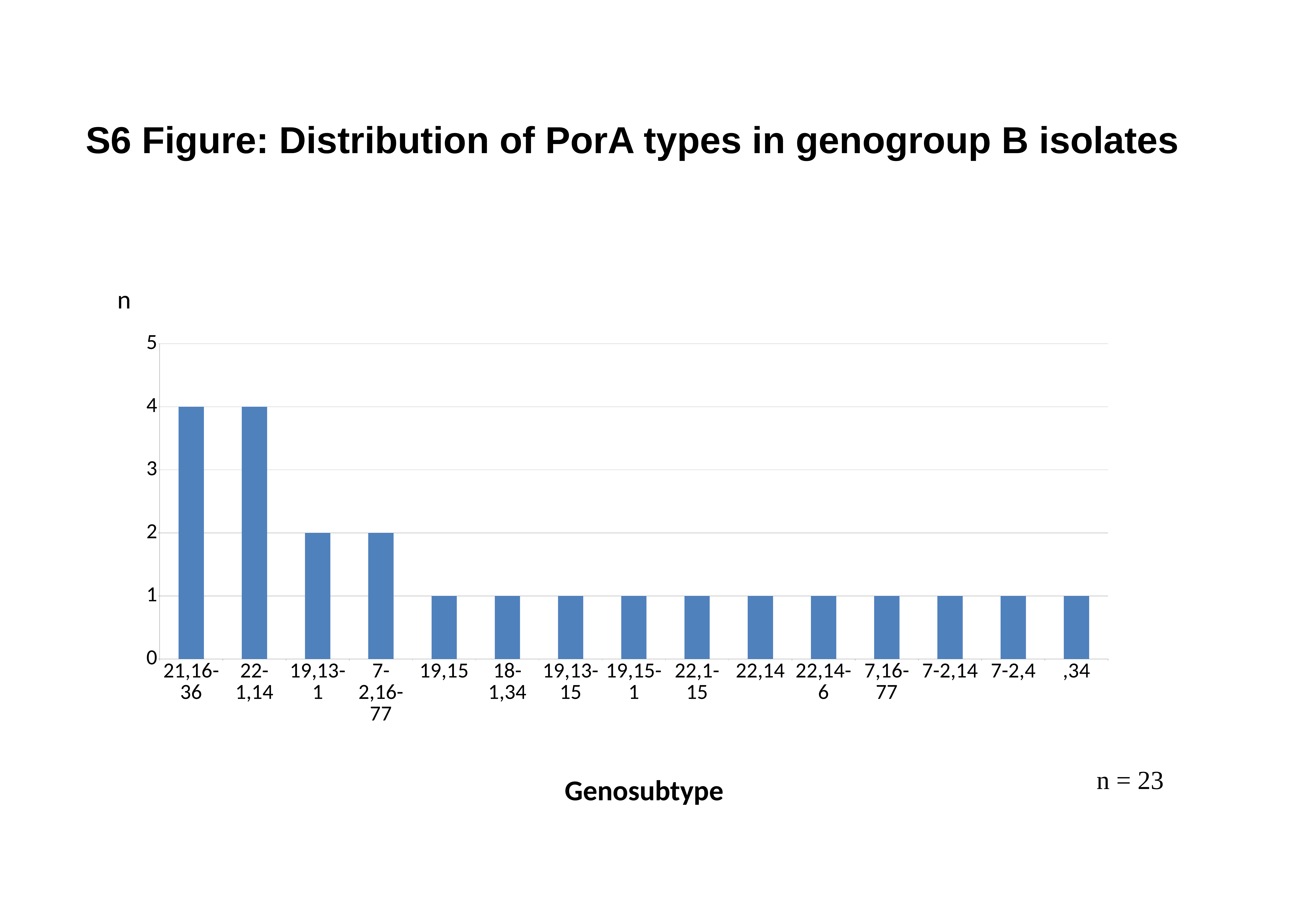

S6 Figure: Distribution of PorA types in genogroup B isolates
### Chart
| Category | |
|---|---|
| 21,16-36 | 4.0 |
| 22-1,14 | 4.0 |
| 19,13-1 | 2.0 |
| 7-2,16-77 | 2.0 |
| 19,15 | 1.0 |
| 18-1,34 | 1.0 |
| 19,13-15 | 1.0 |
| 19,15-1 | 1.0 |
| 22,1-15 | 1.0 |
| 22,14 | 1.0 |
| 22,14-6 | 1.0 |
| 7,16-77 | 1.0 |
| 7-2,14 | 1.0 |
| 7-2,4 | 1.0 |
| ,34 | 1.0 |Genosubtype

Supplement: S6 Fig — (PPTX) [file pone.0247991.s006.pptx]
