# Supplementary material for: Oropharyngeal meningococcal carriage in children and adolescents, a single center study in Buenos Aires, Argentina
Source: PLoS One. 2021 Mar 29;16(3):e0247991. doi: 10.1371/journal.pone.0247991 (PMC8006983; doi:10.1371/journal.pone.0247991)
Supplement: S7 Fig — (PPTX) [file pone.0247991.s007.pptx]

## Slide 1
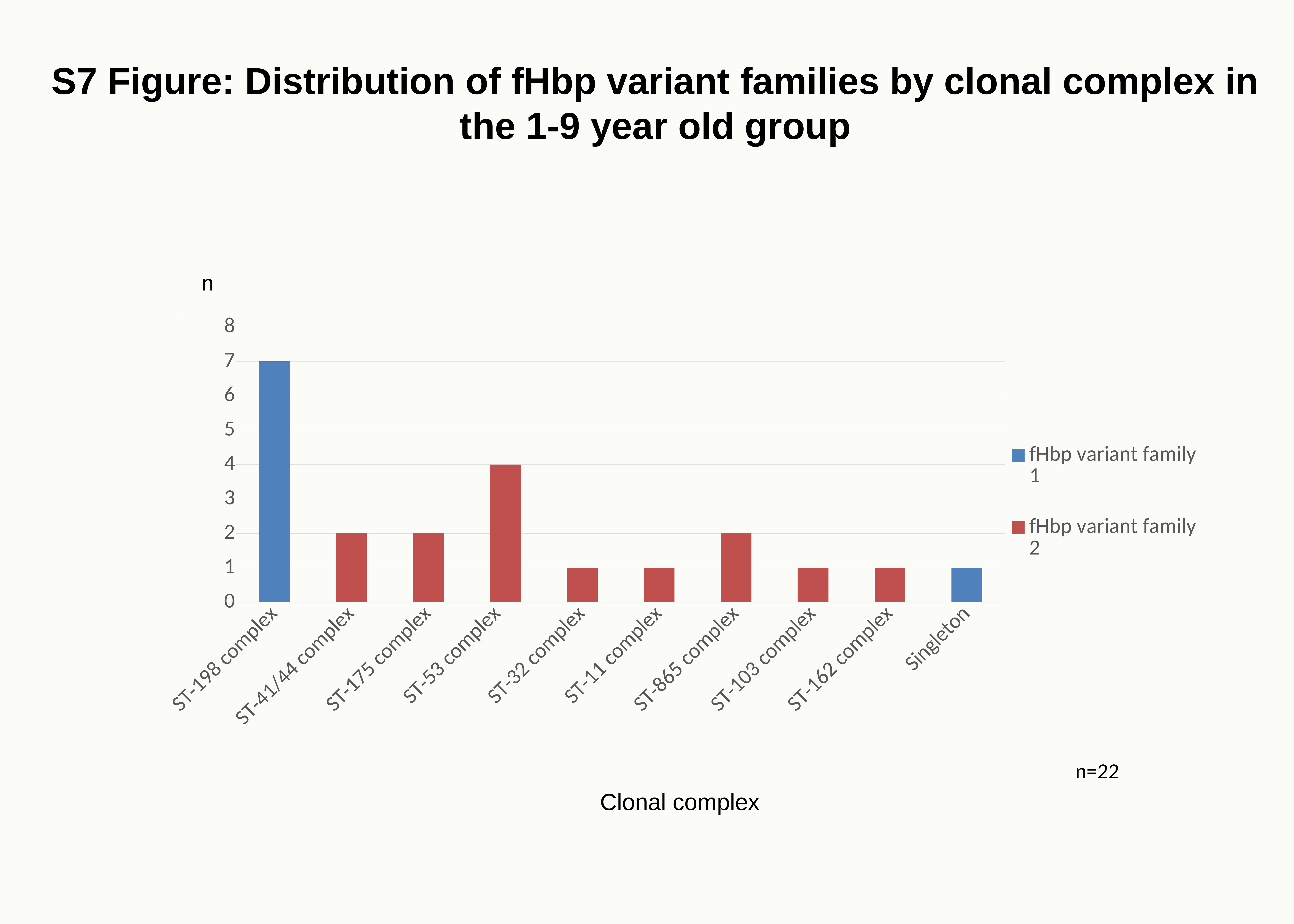

S7 Figure: Distribution of fHbp variant families by clonal complex in the 1-9 year old group
n
### Chart
| Category | fHbp variant family 1 | fHbp variant family 2 |
|---|---|---|
| ST-198 complex | 7.0 | 0.0 |
| ST-41/44 complex | 0.0 | 2.0 |
| ST-175 complex | 0.0 | 2.0 |
| ST-53 complex
 | 0.0 | 4.0 |
| ST-32 complex | 0.0 | 1.0 |
| ST-11 complex | 0.0 | 1.0 |
| ST-865 complex | 0.0 | 2.0 |
| ST-103 complex | 0.0 | 1.0 |
| ST-162 complex | 0.0 | 1.0 |
| Singleton | 1.0 | 0.0 |n=22
