# Supplementary material for: Oropharyngeal meningococcal carriage in children and adolescents, a single center study in Buenos Aires, Argentina
Source: PLoS One. 2021 Mar 29;16(3):e0247991. doi: 10.1371/journal.pone.0247991 (PMC8006983; doi:10.1371/journal.pone.0247991)
Supplement: S1 Table — (DOCX) [file pone.0247991.s009.docx]

**Suplementary Table 1. Oligonucleotides used for *N. meningitidis* molecular typing**

**Identification and genogrouping**

| **Oligonucleotide** | **Sequence (5´to 3¨)** | **Gene amplified (genogroup)** | **Ref** |
| --- | --- | --- | --- |
|  |  |  |  |
| 98 - 6 | GCTGGCGCCGCTGGCAACAAAATTC | *crg*A | 1 |
| 98 - 10 | CTTCTGCAGATTGCGGCGTGCCGT |  |  |
| *ctr*A F | ATGCGGTGGCTGCGGTAGGT | *ctr*A | 3 |
| *ctr*A R | CCGGCGAGAACACAAACGACAAG |  |  |
| HC344 | GGATTGGACGAGCGAGAC | *cnl* | 4 |
| GH26R | GGTCGTCTGAAAGCTTGCCTTGCTC |  |  |
| 98-28 | CGCAATAGGTGTATATATTCTTCC | *orf-2* (A) | 1 |
| 98-29 | CTGAATAGTTTCGTATGCCTTCTT |  |  |
| 98 - 19 | GGATCATTTCAGTGTTTTCCACCA | *sia*D (B) | 1 |
| 98 - 20 | GCATGCTGGAGGAATAAGCATTAA |  |  |
| 98 - 17 | TCAAATGAGTTTGCGAATAGAAGGT | *sia*D (C) | 1 |
| 98 - 18 | CAATCACGATTTGCCCAATTGAC |  |  |
| 98 - 32 | CAGAAAGTGAGGGATTTCCATA | *sia*D (W) | 1 |
| 98 - 33 | CACAACCATTTTCATTATAGTTACTGT |  |  |
| 98 - 34 | CTCAAAGCGAAGGCTTTGGTTA | *sia*D (Y) | 1 |
| 98 - 35 | CTGAAGCGTTTTCATTATAATTGCTAA |  |  |
| UR | TTGTCGCGGATTTGCAACTA | *ctr*A (E, X, Z) | 2 |
| 29EF2 | ATTACGCTGACGGCATGTGGA | *ctr*A (E) |  |
| ZF | TATGCGGTGCTGTTCGCTATG | *ctr*A (X) |  |
| XF3 | GTCTTTGTATAAGGCCCAAG | *ctr*A (Z) |  |

**MLST PCR amplification**

| **Oligonucleotide** | **Sequence** | **Gene** | **Ref** |
| --- | --- | --- | --- |
| abcZ-P1C | TGTTCCGCTTCGACTGCCAAC | *abcZ* | 5 |
| abcZ-P2C | TCCCCGTCGTAAAAAACAATC |  |  |
| adk-P1B | CCAAGCCGTGTAGAATCGTAAACC | *adk* | 5 |
| adk-P2B | TGCCCAATGCGCCCAATAC |  |  |
| aroE-P1B | TTTGAAACAGGCGGTTGCGG | *aroE* | 5 |
| aroE-P2B | CAGCGGTAATCCAGTGCGAC |  |  |
| fumC-P1B | TCCCCGCCGTAAAAGCCCTG | *fumC* | 5 |
| fumC-P2B | GCCCGTCAGCAAGCCCAAC |  |  |
| gdh-P1B | CTGCCCCCGGGGTTTTCATCT | *gdh* | 5 |
| gdh-P2B | TGTTGCGCGTTATTTCAAAGAAGG |  |  |
| pdhC-P1B | CCGGCCGTACGACGCTGAAC | *pdhC* | 5 |
| pdhC-P2B | GATGTCGGAATGGGGCAAACA |  |  |
| pgm-P1 | CTTCAAAGCCTACGACATCCG | *pgm* | 5 |
| pgm-P2 | CGGATTGCTTTCGATGACGGC |  |  |

**MLST sequencing**

| **Oligonucleotide** | **Sequence (5´-3´)** | **Gene** | **Ref** |
| --- | --- | --- | --- |
| abcZ-S1A (P1A) | AATCGTTTATGTACCGCAGR | *abcZ* | 5 |
| abcZ-S2 | GAGAACGAGCCGGGATAGGA |  |  |
| adk-S1A | AGGCWGGCACGCCCTTGG | *adk* | 5 |
| adk-S2 | CAATACTTCGGCTTTCACGG |  |  |
| aroE-S1A | GCGGTCAAYACGCTGRTK | *aroE* | 5 |
| aroE-S2 | ATGATGTTGCCGTACACATA |  |  |
| fumC-S1 | TCCGGCTTGCCGTTTGTCAG | *fumC* | 5 |
| fumC-S2 | TTGTAGGCGGTTTTGGCGAC |  |  |
| gdh-S3 | CCTTGGCAAAGAAAGCCTGC | *gdh* | 5 |
| gdh-S4C | RCGCACGGATTCATRYGG |  |  |
| pdhC-S1 | TCTACTACATCACCCTGATG | *pdhC* | 5 |
| pdhC-S2 | ATCGGCTTTGATGCCGTATTT |  |  |
| pgm-S1 | CGGCGATGCCGACCGCTTGG | *pgm* | 5 |
| pgm-S2A | GGTGATGATTTCGGTYGCRCC |  |  |

**PorA, fHbp, NHBA and NadA typing. PCR amplification**

| **Oligonucleotide** | **Sequence (5´-3´)** | **Gene** | **Ref** |
| --- | --- | --- | --- |
| P1 | GCGGCCGTTGCCGATGTCAGCC | *porA* | 7 |
| P2 | GCGGCATTAATTTGAGTGTAGTTGCC |  |  |
| gna1870F | TGACCTGCCTCATTGATGC | *fHbp* | 9 |
| gna1870R | CGGTAAATTATCGTGTTCGGACGGC |  |  |
| NadA-A | GTGGACGTACTCGACTACGAAGG | *nadA* | 10 |
| NadA-B | CGAGGCGATTGTCAAACCGTTC |  |  |
| NHBA F | GGCGTTCAGACGGCATATTTTTACA | *nhba* | 9 |
| NHBA R | GGTTTATCAACTGATGCGGACTTGA |  |  |

**PorA, fHbp, NHBA and NadA typing. Sequencing**

| **Oligonucleotide** | **Sequence (5´-3´)** | **Gene** | **Ref** |
| --- | --- | --- | --- |
| P1 | GCGGCCGTTGCCGATGTCAGCC | *porA* | 7 |
| 8Ua | TCCGTACGCTACGATTCTCC |  | 6 |
| P2 | GCGGCATTAATTTGAGTGTAGTTGCC |  | 7 |
| 103L^a^ | AACGGATACGTCTTGCTC |  | 8 |
| 122L^a^ | GGCGAGATTCAAGCCGCC |  | 6 |
| gna1870S2 | CAAATCGAAGTGGACGGGCAG | *fHbp* | 11 |
| gna1870S3 | TGTTCGATTTTGCCGTTTCCCTG |  | 9 |
| gna1870R2^b^ | CGTGCCGTCGTGTCCTAG |  | 11 |
| NHBA F | GGCGTTCAGACGGCATATTTTTACA | *nhba* | 9 |
| NHBA R | GGTTTATCAACTGATGCGGACTTGA |  |  |

| ^a^Alternative primer |
| --- |
| ^b^ Alternative primer for PCR failures due to deletion in gna1870R primer binding site |

**References**

[1] Taha MK. Simultaneous approach for nonculture PCR-based identification and serogroup prediction of Neisseria meningitidis. J Clin Microbiol 2000 Feb;38(2):855-7.

[2]Bennett DE, Mulhall RM, Cafferkey MT. PCR-based assay for detection of Neisseria meningitidis capsular serogroups 29E, X, and Z. J Clin Microbiol 2004 Apr;42(4):1764-5.

[3] Malcolm Guiver, Ray Borrow, John Marsh, Stephen J. Gray, Edward B. Kaczmarski, David Howells, Paul Boseley, Andrew J. FoxEvaluation of the Applied Biosystems automated Taqmanpolymerase chain reaction system for the detection of meningococcal DNA.FEMS Immunology and Medical Microbiology 2000 28 173-179

[4]Claus H, Maiden MC, Maag R, Frosch M, Vogel U. Many carried meningococci lack the genes required for capsule synthesis and transport. Microbiology 2002 Jun;148(Pt 6):1813-9.

[5] Maiden MC, Bygraves JA, Feil E, Morelli G, Russell JE, Urwin R, et al. Multilocus sequence typing: a portable approach to the identification of clones within populations of pathogenic microorganisms. Proc Natl Acad Sci U S A 1998 Mar 17;95(6):3140-5.

[6] Maiden, M. C., J. A. Bygraves, J. McCarvil, and I. M. Feavers. 1992. Identification of meningococcal serosubtypes by polymerase chain reaction. J Clin Microbiol 30:2835-41.

[7] Saunders, N. B., W. D. Zollinger, and V. B. Rao. 1993. A rapid and sensitive PCR strategy employed for amplification and sequencing of porA from a single colony-forming unit of Neisseria meningitidis. Gene 137:153-62.

[8]Suker, J., I. M. Feavers, M. Achtman, G. Morelli, J. F. Wang, and M. C. Maiden. 1994. The porA gene in serogroup A meningococci: evolutionary stability and mechanism of genetic variation. Mol Microbiol 12:253-65.

[9] Jacobsson, S., S. Thulin, P. Molling, M. Unemo, M. Comanducci, R. Rappuoli, and P. Olcen. 2006. Sequence constancies and variations in genes encoding three new meningococcal vaccine candidate antigens. Vaccine 24:2161-8.

[10] Stefania Bambini, Alessandro Muzzi, Per Olcen, Rino Rappuoli a,

Mariagrazia Pizzaa, Maurizio Comanducci. Distribution and genetic variability of three vaccine components in a panel of strains representative of the diversity of serogroup B meningococcus. Vaccine 2009 (27) 2794–2803

[11] Lucidarme J, Comanducci M, Findlow J, Gray SJ, Kaczmarski EB, Guiver M, et al. Characterization of fHbp, nhba (gna2132), nadA, porA, sequence type (ST), and genomic presence of IS1301 in group B meningococcal ST269 clonal complex isolates from England and Wales. J Clin Microbiol 2009 Nov;47(11):3577-85
