# Supplementary material for: Oropharyngeal meningococcal carriage in children and adolescents, a single center study in Buenos Aires, Argentina
Source: PLoS One. 2021 Mar 29;16(3):e0247991. doi: 10.1371/journal.pone.0247991 (PMC8006983; doi:10.1371/journal.pone.0247991)
Supplement: S2 Table — (DOCX) [file pone.0247991.s010.docx]

| **Supplementary Table 2. Molecular data.** | | | | | | | | | | | |
| --- | --- | --- | --- | --- | --- | --- | --- | --- | --- | --- | --- |
| **Isolate** | **Genogroup** | **Age group (y.o)** | **fHbp peptide (Novartis nomenclature)** | | | **NHBA** | | **porA** | | **Sequence type** | **Clonal Complex** |
|  |  |  | **Allele** | **Variant family** | **Novartis variant** | **Allele** | **Peptide variant** | **VR1** | **VR2** |  |  |
| 0013 | W | 10-19 | 22 | 2 | 2.22 | NA | NA | 5 | 2 | 11 | ST-11 complex |
| 0053 | cnl | 10-19 | 4 | 1 | 1.4 | 35 | 10 | 18 | 25-44 | 823 | ST-198 complex |
| 0055 | cnl | 1-9 | 4 | 1 | 1.4 | 35 | 10 | 19 | 14 | 13652 | ST-198 complex |
| 0062 | cnl | 10-19 | 102 | 2 | 2.102 | NA | NA | 7 | 30-2 | 53 | ST-53 complex |
| 0078 | C | 10-19 | 24 | 2 | 2.24 | NA | NA | 7-2 | 13-1 | 278 | ST-35 complex |
| 0130 | Z | 1-9 | 25 | 2 | 2.25 | 39 | 13 | 5-1 | 2-2 | 13624 | ST-103 complex |
| 0174 | cnl | 10-19 | 16 | 2 | 2.16 | 44 | 145 | 18-1 | 3-4 | 1136 | ST-1136 complex |
| 0185 | cnl | 10-19 | 4 | 1 | 1.4 | 35 | 10 | 18 | 25-44 | 823 | ST-198 complex |
| 0209 | cnl | 10-19 | 4 | 1 | 1.4 | 77 | 476 | 22 | 14 | 3876 | ST-198 complex |
| 0230 | B | 1-9 | NA | NA | NA | NA | NA | 22 | 14 | 6987 | ST-41/44 complex |
| 0270 | cnl | 1-9 | 4 | 1 | 1.4 | 35 | 10 | 22 | 14 | 3876 | ST-198 complex |
| 0282 | Y | 10-19 | 119 | 2 | 2.119 | NA | NA | 22-1 | 14 | 13638 | NA |
| 0283 | cnl | 10-19 | NA | NA | NA | 35 | 10 | 22 | 14 | 3876 | ST-198 complex |
| 0342 | cnl | 1-9 | NA | NA | NA | 44 | 145 | 22 | 14 | 1136 | ST-1136 complex |
| 0441 | OG | 1-9 | NA | NA | NA | NA | NA | 22 | 26 | 5770 | ST-175 complex |
| 0475 | cnl | 1-9 | 4 | 1 | 1.4 | 35 | 10 | 22 | 14 | 3876 | ST-198 complex |
| 0498 | cnl | 10-19 | NA | NA | NA | NA | NA | 18-1 | NA | 13639 | ST-1136 complex |
| 0520 | cnl | 1-9 | 4 | 1 | 1.4 | NA | NA | 5-24 | NA | 823 | ST-198 complex |
| 0534 | C | 10-19 | 19 | 2 | 2.19 | 7 | 9 | 18-7 | 9 | 5770 | ST-175 complex |
| 0537 | cnl | 10-19 | 4 | 1 | 1.4 | NA | NA | 18 | 25-44 | 823 | ST-198 complex |
| 0538 | cnl | 10-19 | 4 | 1 | 1.4 | NA | NA | 18 | 25-44 | 823 | ST-198 complex |
| 0553 | OG | 1-9 | 19 | 2 | 2.19 | NA | NA | 18-7 | 9 | 5770 | ST-175 complex |
| 0572 | OG | 10-19 | 77 | 2 | 2.77 | NA | NA | 22-1 | 14 | 35 | ST-35 complex |
| 0584 | cnl | 10-19 | 4 | 1 | 1.4 | NA | NA | 22 | 14 | 3876 | ST-198 complex |
| 0598 | B | 1-9 | NA | NA | NA | NA | NA | NA | NA | 35 | ST-35 complex |
| 0601 | B | 10-19 | NA | NA | NA | NA | NA | NA | NA | 8869 | ST-41/44 complex |
| 0624 | B | 1-9 | 119 | 2 | 2.119 | 15 | 24 | 21 | 16-36 | 3327 | ST-865 complex |
| 0647 | OG | 10-19 | 101 | 2 | 2.101 | NA | NA | 19 | 13-9 | 13658 | NA |
| 0708 | cnl | 10-19 | 102 | 2 | 2.102 | NA | NA | 7 | 30 | 13698 | ST-53 complex |
| 0756 | W | 1-9 | 22 | 2 | 2.22 | NA | NA | 5 | 2 | 11 | ST-11 complex |
| 0779 | cnl | 1-9 | 4 | 1 | 1.4 | NA | NA | 5-24 | NA | 823 | ST-198 complex |
| 0784 | B | 1-9 | 119 | 2 | 2.119 | NA | NA | 21 | 16-36 | 3327 | ST-865 complex |
| 0803 | cnl | 1-9 | 4 | 1 | 1.4 | 35 | 10 | 18 | 25-44 | 823 | ST-198 complex |
| 0806 | B | 1-9 | 19 | 2 | 2.19 | NA | NA | 7-2 | 14 | 13660 | ST-41/44 complex |
| 0837 | W | 10-19 | 16 | 2 | 2.16 | 19 | 21 | 22-1 | 14 | 35 | ST-35 complex |
| 0838 | W | 1-9 | NA | NA | NA | 723 | 657 | 22-1 | 14 | 35 | ST-35 complex |
| 0844 | W | 10-19 | 19 | 2 | 2.19 | 7 | 9 | NA | 9 | 13646 | ST-1136 complex |
| 0854 | B | 1-9 | 105 | 2 | 2.105 | NA | NA | 7 | 16-77 | 1880 | ST-32 complex |
| 0861 | B | 1-9 | 21 | 2 | 2.21 | 11 | 20 | 7-2 | 4 | 10817 | ST-162 complex |
| 0862 | cnl | 10-19 | 4 | 1 | 1.4 | 35 | 10 | 5-24 | NA | 13640 | ST-198 complex |
| 0899 | Y | 1-9 | 19 | 2 | 2.19 | 7 | 9 | 18-7 | 9 | 5770 | ST-175 complex |
| 0915 | B | 10-19 | 105 | 2 | 2.105 | NA | NA | 7-2 | 16-77 | 13661 | ST-32 complex |
| 0964 | cnl | 10-19 | 4 | 1 | 1.4 | 35 | 10 | 22 | 14 | 3876 | ST-198 complex |
| 0967 | cnl | 1-9 | 102 | 2 | 2.102 | 65 | 58 | 7 | 30-8 | 13662 | ST-53 complex |
| 0972 | Y | 10-19 | 25 | 2 | 2.25 | 335 | 145 | 18-1 | 30-11 | 6519 | ST-23 complex |
| 0984 | W | 10-19 | 119 | 2 | 2.119 | NA | NA | 21 | 16-36 | 13647 | NA |
| 1024 | cnl | 1-9 | 102 | 2 | 2.102 | NA | NA | 7 | 30 | 13648 | ST-53 complex |
| 1033 | cnl | 1-9 | 14 | 1 | 1.14 | NA | NA | 22 | 14-16 | 13699 | NA |
| 1081 | B | 10-19 | 119 | 2 | 2.119 | NA | NA | 21 | 16-36 | 3327 | ST-865 complex |
| 1096 | cnl | 1-9 | 4 | 1 | 1.4 | NA | NA | 22 | 26-4 | 13640 | ST-198 complex |
| 1109 | B | 10-19 | 19 | 2 | 2.19 | NA | NA | 18-1 | 34 | 409 | ST-41/44 complex |
| 1122 | B | 1-9 | NA | NA | NA | 19 | 21 | 22-1 | 14 | 35 | ST-35 complex |
| 1124 | B | 1-9 | 19 | 2 | 2.19 | 938 | 890 | NA | 34 | 409 | ST-41/44 complex |
| 1125 | W | 10-19 | 22 | 2 | 2.22 | 17 | 29 | 5 | 2 | 11 | ST-11 complex |
| 1155 | cnl | 1-9 | 102 | 2 | 2.102 | NA | NA | 7 | 30 | 53 | ST-53 complex |
| 1171 | cnl | 10-19 | 102 | 2 | 2.102 | 65 | 58 | 7 | 30-2 | 53 | ST-53 complex |
| 1196 | B | 1-9 | 16 | 2 | 2.16 | 3 | 20 | 7-2 | 16-77 | 1880 | ST-32 complex |
| 1223 | cnl | 1-9 | NA | NA | NA | NA | NA | NA | NA | 1136 | ST-1136 complex |
| 1224 | cnl | 1-9 | NA | NA | NA | NA | NA | 22 | 14-21 | 1136 | ST-1136 complex |
| 1225 | cnl | 1-9 | NA | NA | NA | NA | NA | 5 | 2 | 1136 | ST-1136 complex |
| 1236 | OG | 1-9 | NA | NA | NA | NA | NA | 19 | 13-9 | 13700 | NA |
| 1244 | cnl | 10-19 | 4 | 1 | 1.4 | 35 | 10 | 22 | 14-3 | 3876 | ST-198 complex |
| 1245 | B | 10-19 | NA | NA | NA | NA | NA | 22 | 1-15 | 13649 | ST-750 complex |
| 1261 | cnl | 10-19 | 4 | 1 | 1.4 | 35 | 10 | 22 | 14 | 13663 | ST-198 complex |
| 1269 | B | 10-19 | 19 | 2 | 2.19 | 17 | 29 | 19 | 13-1 | 44 | ST-41/44 complex |
| 1291 | cnl | 10-19 | 4 | 1 | 1.4 |  |  | 22 | 14 | 3876 | ST-198 complex |
| 1299 | Y | 10-19 | 1146 | 1 | NA | 7 | 9 | 5-2 | NA | 6626 | ST-175 complex |
| 1307 | cnl | 10-19 | 4 | 1 | 1.4 | 35 | 10 | 22 | 14 | 3876 | ST-198 complex |
| 1330 | OG | 10-19 | 19 | 2 | 2.19 | 7 | 9 | 18-7 | 9 | 5770 | ST-175 complex |
| 1332 | Z | 10-19 | 102 | 2 | 2.102 | 35 | 10 | 19 | 13-9 | 13641 | NA |
| 1338 | cnl | 10-19 | NA | NA | NA | 44 | 145 | 18-4 | 25 | 1136 | ST-1136 complex |
| 1346 | cnl | 10-19 | 4 | 1 | 1.4 | 35 | 10 | 5-11 | NA | 823 | ST-198 complex |
| 1351 | cnl | 10-19 | 1147 | 1 | NA | 35 | 10 | 22-28 | 14 | 3876 | ST-198 complex |
| 1354 | Y | 10-19 | 25 | 2 | 2.25 | 81 | 470 | 18-1 | 30 | 6519 | ST-23 complex |
| 1404 | cnl | 10-19 | 4 | 1 | 1.4 | 35 | 10 | 22 | 14 | 3876 | ST-198 complex |
| 1406 | B | 10-19 | 16 | 2 | 2.16 | 19 | 21 | 22-1 | 14 | 35 | ST-35 complex |
| 1425 | cnl | 10-19 | 4 | 1 | 1.4 | 35 | 10 | 5-11 | NA | 823 | ST-198 complex |
| 1428 | cnl | 10-19 | 4 | 1 | 1.4 | 35 | 10 | 22 | 14 | 823 | ST-198 complex |
| 1436 | B | 10-19 | 16 | 2 | 2.16 | 19 | 21 | 22-1 | 14 | 35 | ST-35 complex |
| 1438 | B | 10-19 | 19 | 2 | 2.19 | 17 | 29 | 19 | 13-1 | 13642 | ST-41/44 complex |
| 1470 | cnl | 10-19 | NA | NA | NA | NA | NA | 18-4 | 25 | 13643 | ST-1136 complex |
| 1493 | Z | 10-19 | 21 | 2 | 2.21 | NA | NA | 7-2 | 9 | 13701 | NA |
| 1526 | W | 10-19 | 16 | 2 | 2.16 | 19 | 21 | 22-1 | 14 | 7479 | ST-35 complex |
| 1530 | C | 10-19 | 168 | 3 | 3.168 | 92 | 118 | 19-2 | 13-1 | 461 | ST-461 complex |
| 1543 | B | 10-19 | 1 | 1 | 1.1 | NA | NA | 19 | 15 | 33 | ST-32 complex |
| 1573 | B | 10-19 | 24 | 2 | 2.24 | 19 | 21 | 19 | 15-1 | 11827 | ST-35 complex |
| 1594 | cnl | 10-19 | 4 | 1 | 1.4 | 35 | 10 | 22 | 14 | 3876 | ST-198 complex |
| 1601 | OG | 10-19 | 14 | 1 | 1.14 | 315 | 183 | 22 | 14-20 | 12958 | NA |
| 1602 | cnl | 10-19 | 4 | 1 | 1.4 | 35 | 10 | 22 | NA | 13640 | ST-198 complex |
| 1606 | C | 10-19 | 19 | 2 | 2.19 | 37 | 1 | 5-1 | 2-19 | 2731 | ST-41/44 complex |
| 1622 | cnl | 10-19 | NA | NA | NA | 44 | 145 | 18-4 | 25 | 1136 | ST-1136 complex |
| 1624 | B | 10-19 | 119 | 2 | 2.119 | 15 | 24 | 21 | 16-36 | 13664 | NA |
| 1627 | cnl | 10-19 | 4 | 1 | 1.4 | 1452 | 1307 | 18-7 | 9 | 823 | ST-198 complex |
| 1642 | Y | 10-19 | 21 | 2 | 2.21 | 9 | 6 | 21 | 16 | 1466 | ST-174 complex |
| 1643 | cnl | 10-19 | 161 | 2 | 2.161 | 1 | 2 | 18-1 | 34 | 13625 | ST-41/44 complex |
| 1669 | cnl | 10-19 | 4 | 1 | 1.4 | 35 | 10 | NA | NA | 13665 | ST-198 complex |
| 1679 | B | 10-19 | 19 | 2 | 2.19 | 17 | 29 | 19 | 13-15 | 44 | ST-41/44 complex |
| 1680 | Y | 10-19 | NA | NA | NA | 275 | 53 | 21 | 4 | 13644 | NA |
| 1683 | OG | 10-19 | 16 | 2 | 2.16 | NA | NA | 18-1 | NA | 13666 | NA |
| 1703 | OG | 10-19 | 13 | 1 | 1.13 | 15 | 24 | 5 | 2 | 7983 | ST-60 complex |
| 1709 | cnl | 10-19 | 4 | 1 | 1.4 | 35 | 10 | 22-28 | 14 | 3876 | ST-198 complex |
| 1728 | B | 10-19 | 16 | 2 | 2.16 | 19 | 21 | 22-1 | 14 | 35 | ST-35 complex |
| 1731 | B | 10-19 | 203 | 2 | 2.203 | 934 | 886 | 22 | 14-6 | 1880 | ST-32 complex |
| 1734 | cnl | 10-19 | 4 | 1 | 1.4 | 35 | 10 | 22-3 | 26 | 3876 | ST-198 complex |
| 1750 | Y | 10-19 | 21 | 2 | 2.21 | 9 | 6 | 21 | 16 | 1466 | ST-174 complex |
| NA Not available | | | | | |  |  |  |  |  |  |
