# Supplementary material for: Oropharyngeal meningococcal carriage in children and adolescents, a single center study in Buenos Aires, Argentina
Source: PLoS One. 2021 Mar 29;16(3):e0247991. doi: 10.1371/journal.pone.0247991 (PMC8006983; doi:10.1371/journal.pone.0247991)
Supplement: S1 File — (DOCX) [file pone.0247991.s011.docx]

***Data Form:***

| **Nm carriage Study** | | | | | |
| --- | --- | --- | --- | --- | --- |
| CODE ID: | | | Sex: | | |
| Age (in months): | | | | | |
| Day and Sample Time: | | | | | |
| Where do you live? (Province and Municipality) | | | | | |
| How many people share the home? | | | | | |
| How many rooms are in the house? | | | | | |
| Crowded housing conditions (Simple/Critical) | | | | | |
| Maternal Education: | primary (7 years schooling), | Complete: |  | Incomplete: |  |
|  | secondary (11 years) | Complete: |  | Incomplete: |  |
|  | tertiary/university (>11 years) | Complete: |  | Incomplete: |  |
| Did the subject received antibiotics in the past 3 months? | | | | | |
| Active Smoking? (yes/no) | | Passive Smoking? (yes/no) | | | |
| Did the subject assist to Kindergarten/School/Secondary/University? (yes/no) | | | | | |
| Did the subject go to Pubs/Nightclubs? (yes/no): How many times per week? | | | | | |
| Did the subject go to club/art class outside the School time? (yes/no) How many times per week? | | | | | |
| ***Microbiology data:*** | | | | | |
| Cultive result: | | | | | |

***Encuesta:***

| **Estudio de portación de Nm** | | | | | | |
| --- | --- | --- | --- | --- | --- | --- |
| Código de Identificación: | | | Sexo: | |  |  |
| Edad (en meses): | | | | | | |
| Día y hora de la toma de muestra: | | | | | | |
| Donde vive? (Provincia y Municipio) | | | | | | |
| Cuantas personas comparten la casa? | | | | | | |
| Cuántas habitaciones hay en la casa? | | | | | | |
| Condiciones de hacinamiento (Simple/Crítico) | | | | | | |
| Educación materna: | Primaria (7 años), | Completo: |  | Incompleto: | |  |
|  | Secundaria (11 años) | Completo: |  | Incompleto: | |  |
|  | Terciaria/Universitaria (>11 años) | Completo: |  | Incompleto: | |  |
| El sujeto recibió antibióticos en los 3 meses previos? | | | | | | |
| Fumador activo? (si/no) | | Tabaquismo pasivo? (si/no) | | | | |
| El sujeto asiste a jardín de infantes/escuela/secundaria/universidad? (si/no) | | | | | | |
| El sujeto frecuenta bares/clubes nocturnos? (si/no): Cuántas veces por semana? | | | | | | |
| El sujeto realiza actividades extra escolares (deportes/clases de arte/danza)? (si/no) Cuántas veces por semana? | | | | | | |
| ***Datos microbiológicos:*** | | | | | | |
| Resultado de cultivo: | | | | | | |
